# Fine Structure of Glycosaminoglycans from Fresh and Decellularized Porcine Cardiac Valves and Pericardium

**DOI:** 10.1155/2012/979351

**Published:** 2012-02-22

**Authors:** Antonio Cigliano, Alessandro Gandaglia, Antonio Junior Lepedda, Elisabetta Zinellu, Filippo Naso, Alessandra Gastaldello, Paola Aguiari, Pierina De Muro, Gino Gerosa, Michele Spina, Marilena Formato

**Affiliations:** ^1^Dipartimento di Scienze Fisiologiche, Biochimiche e Cellulari, Università di Sassari, Viale Muroni 25, 07100 Sassari, Italy; ^2^Dipartimento di Scienze Cardiologiche, Toraciche e Vascolari, Università di Padova, Viale Giustiniani 2, 35128 Padova, Italy; ^3^Dipartimento di Scienze Biomediche Sperimentali, Università di Padova, Viale G. Colombo 3, 35131 Padova, Italy

## Abstract

Cardiac valves are dynamic structures, exhibiting a highly specialized architecture consisting of cells and extracellular matrix with a relevant proteoglycan and glycosaminoglycan content, collagen and elastic fibers. Biological valve substitutes are obtained from xenogenic cardiac and pericardial tissues. To overcome the limits of such non viable substitutes, tissue engineering approaches emerged to create cell repopulated decellularized scaffolds. This study was performed to determine the glycosaminoglycans content, distribution, and disaccharides composition in porcine aortic and pulmonary valves and in pericardium before and after a detergent-based decellularization procedure. The fine structural characteristics of galactosaminoglycans chondroitin sulfate and dermatan sulfate were examined by FACE. Furthermore, the mechanical properties of decellularized pericardium and its propensity to be repopulated by in vitro seeded fibroblasts were investigated. Results show that galactosaminoglycans and hyaluronan are differently distributed between pericardium and valves and within heart valves themselves before and after decellularization. The distribution of glycosaminoglycans is also dependent from the vascular district and topographic localization. The decellularization protocol adopted resulted in a relevant but not selective depletion of galactosaminoglycans. As a whole, data suggest that both decellularized porcine heart valves and bovine pericardium represent promising materials bearing the potential for future development of tissue engineered heart valve scaffolds.

## 1. Introduction

Heart valve disease has a deep impact worldwide related with the large number of valvular replacement operations performed every year. Typical valve substitutes are mechanical prostheses and bioprostheses obtained from cardiac-valvulated conduits (aortic and pulmonary root) or pericardial tissue of porcine and bovine origin. Bioprosthetic valves, although associated with a lower risk of thromboembolism with respect to the mechanical ones, possess limited longevity due to dystrophic calcification consequent to glutaraldehyde (GA) treatment used for preventing rejection [1] and suffer for many of the same degenerative processes that afflict native valves [2]. In the last years, tissue engineering (TE) approaches raised in response to limitations associated with tissue and organ transplantation and with the scarcity of available donors. Three are the components essential for a TE substitute: cells, scaffolds (designed to maintain the cells in a three-dimensional environment), and signals that guide the gene expression and ECM production during tissue development [3]. The typical approach relies on the use of acellular matrix xenografts (mostly porcine and bovine) as scaffold that would ultimately become repopulated with cells from the patient [4]. Repopulation of natural matrix xenografts has been proposed for its greater chance of success with respect to that of biopolymeric scaffolds [5, 6]. The surgical procedures, used to date for the implantation of commercially available devices, are represented by the transcatheter aortic valve replacement (TAVI) technique and the open chest classical method. The use of TAVI is indeed emerging especially in patients with severe aortic stenosis and multiple comorbidities that might preclude open chest valve replacements [4]. Most of these expandable grafts are built within pericardial tissue.

The relative amounts and distributions of glycosaminoglycans/proteoglycans (GAGs/PGs) have been reported to be different according to the type of mechanical loading [7, 8]. The knowledge of the composition and distribution of the various GAGs and PGs appears to be essential for understanding the relationship between structure and mechanics of heart valve leaflets [9]. The influence of GAGs and PGs on cell migration, proliferation, and differentiation is well known, besides their role in morphogenesis, angiogenesis, wound healing, immune responses, maintenance of tissue viscoelasticity, and resistance to compression and tension [7, 10, 11].

The maintenance of structural and functional ECM integrity is of primary importance for the performance of the valve substitutes; consequently, their depletion or alteration could be responsible for graft deterioration [12].

GAGs are capable of absorbing a large amount of water within the tissue matrix, due to their high concentration of negative charges and hydrophilicity. This reason led to considering them as fundamental components for the mechanical behavior, given the ability to hydrate the spongiosa layer (decreasing the shear stresses associated with cuspal flexure in valve function), and to absorb compressive forces reducing buckling during flexion [13–16]. Furthermore, their high negative charge may reduce the calcification process by chelating calcium ions. These observations suggest that the loss of the GAGs might dramatically compromise the mechanical function, structure, and/or the onset of dystrophic calcification of bioprosthetic heart valves [17, 18].

A loss of GAGs has been described in both fixed tissues, during the preparation of bioprostheses, and in cryopreserved native tissues [19], with possible consequences on the graft performance. Reports characterizing valve GAGs are largely referred to bioprosthetic valves and tissue-engineered heart valves (TEHVs) [14, 16, 19], whereas only few investigations concerned GAG distribution in the native valve [20–22] and their fine structure. The structural properties of GAGs, such as the extent and pattern of sulfation, the charge density, and the epimerization of their uronic acid moiety are thought to be critical for their function and cellular signaling [23–25]. In vivo each of them can be modulated mediating several biological processes that promote the interaction with different ECM molecules and cells. Recently, we reported the impact of detergent based cell removal on structural components distribution and hydration in aortic and pulmonary heart valve conduits highlighting a relevant depletion of GAGs [26]. This study is focused on the fine structural analysis of the most abundant galactosaminoglycans (GalAGs), that is, the chondroitin sulfate (CS) isomers. The aim of the present study was to characterize the distribution as well as the fine structure of GalAGs in native and decellularized porcine cardiac valves and pericardium due to their extensive use as bioprosthetic material in heart valve replacement. Moreover, we tested the mechanical properties of decellularized-treated pericardium and its propension to be repopulated by in vitro seeded fibroblasts.

## 2. Materials and Methods

### 2.1. Chemicals

Standard preparations of ΔDi-nonS_cs_, 2-acetamido-2-deoxy-3-0-(4-deoxy-*α*-L-threo-hex-4-enopyranosyluronic acid)-4-D-galactose; ΔDi-mono6S, 2-acetamido-2-deoxy-3-0-(4-deoxy-*α*-L-threo-hex-4-enopyranosyluronic acid)-6-O-sulpho-D-galactose; ΔDi-mono4S, 2-acetamido-2-deoxy-3-0-(4-deoxy-*α*-L-threo-hex-4-enopyranosyluronic acid)-4-O-sulpho-D-galactose were all purchased from Seikagaku (Tokyo, Japan); ΔDi-nonS_HA_, 2-acetamido-2-deoxy-3-O-(4-deoxy-*α*-L-threo-hex-4-enopyranosyluronic acid)-4-D-glucose; ΔDi-mono2S, 2-acetamido-2-deoxy-3-O-(4-deoxy-2-O-sulpho-*α*-L-threo-hex-4-enopyranosyluronic acid)-D-galactose; ΔDi-di(2,4)S, 2-acetamido-2-deoxy-3-O-(4-deoxy-2-O-sulfo-*α*-L-threo-hex-4-enopyranosyluronic acid)-4-O-sulfo-D-galactose; ΔDi-di(4,6)S, 2-acetamido-2-deoxy-3-O-(4-deoxy-*α*-L-threo-hex-4-enopyranosyluronic acid)-4,6-O-sulpho-D-galactose were purchased from Dextra Laboratories. Papain from Papaya latex (EC 3.4.22.2), chondroitin ABC lyase from *Proteus vulgaris* (EC 4.2.2.4), chondroitin AC lyase from *Arthrobacter Aurescens* (4.2.2.5), AMAC (>98%), glacial acetic acid, sodium acetate, chloride acid, DMSO (99.9%), sodium cyanoborohydride, cysteine, sodium chloride, and EDTANa_2_ were all obtained from Sigma-Aldrich. Acrylamide and N,N′-methylenebisacrylamide were from BioRad, TEMED (99%), and ammonium persulfate (98%) were purchased from Sigma-Aldrich. DEAE Sephacel was from Amersham Biosciences. All other chemicals used were of analytical reagent grade. 

### 2.2. Harvesting and Tissue Analysis

Porcine hearts from 10–12-month-old pigs with weights ranging from 160 to 180 kg were obtained from a local slaughterhouse. Within 2 hours from death, 12 dissected heart valve conduits, aortic and pulmonary roots (AR and PR), as well as a pool of pericardia (*n* = 6), were harvested and decellularized according to a previously described detergent-based procedure [26]. The pool of pericardial tissue was divided into 2 parts. One was considered as a control and the other subjected to decellularization. The valve conduits were divided into 2 groups: 12 ARs and 12 PRs; for each group 6 samples were considered as control and referred to as NT and 6 samples decellularized with Triton X-100 and Sodium Cholate and referred to as TriCol.

NT samples were rinsed in isotonic saline solution and immediately processed. Briefly, TriCol samples were extracted in hypotonic solution using 1% (w/v) Triton X-100 in presence of protease inhibitors (PI) at 4°C. Following treatment in hypertonic conditions, tissues are extracted in 10 mM sodium cholate at room temperature [26, 27]. After extensive washings for detergent removal, each AR and PR sample was cut into three zones corresponding to: aortic and pulmonary wall, sinus area, and leaflet. Fatty adherences were removed from pericardia by gentle peeling. Wet weight of each valve conduit component and pericardial sample was determined after gently blotting with filter paper (Whatman filter 147 paper number 3). Valve and pericardial minced tissues were dehydrated with 20 volumes of acetone at 4°C for 24 h, defatted with 20 volumes of chloroform: methanol (2 : 1, vol/vol) at 4°C for 24 h, dried for 24 h at 60°C after centrifugation at 3300 ×g for 15 minutes, and finally weighed (dry-defatted tissue (DDT) weight). 

### 2.3. Extraction and Purification of Total GAGs

DDTs (100 mg) were rehydrated for 24 h at 4°C in 0.1 M sodium acetate, pH 6.0, containing 5 mM cysteine, and 5 mM ethylenediaminetetraacetic acid (37 volumes per gram of DDT). Then, papain (0.3 U/mg of DDT) was added to the mixture, which was incubated at 56°C for 48 h under mild agitation. The digest was clarified by centrifugation (9000 ×g for 20 min at +4°C). Digest supernatant was loaded on a (diethylamino)ethyl-cellulose column (0.7 × 6 cm, 2.3 mL), equilibrated with 50 mM sodium acetate, pH 6.0. The column was then washed with 50 mL of the same buffer and eluted with a two-step salt gradient (0.55 and 1.0 M NaCl). Fractions of 1 mL were collected and assayed for hexuronate content by the method of Bitter and Muir, using glucuronolactone as a standard [28]. Total GAG concentrations were estimated by summing the contents of the two elution steps. Fractions containing GAGs from both elution steps were pooled and precipitated using 4 volumes of cold absolute ethanol. The mixture was left overnight at −20°C, and the precipitate was separated by centrifugation, washed twice with ethanol, and then dried.

### 2.4. Fluorophore-Assisted Carbohydrate Electrophoresis (FACE) Analysis

FACE was used to analyze the quantity and fine structure of the different GAG classes, mainly HA and chondroitin/dermatan sulfates (CS/DS). This technology can quickly provide characteristics about the sulfation and iduronation patterns of GalAGs, providing clues to the identification of particular PGs within valve tissues. The enzymes used for the degradation of the CS isomers belong to the family of chondro/dermato lyases. Chondroitinase ABC cleaves the glycosidic bond between hexosamine and glucuronate (GlcA) so degrading hyaluronan (HA) and all chondroitin sulfate isomers (CS A, CS C, and DS), producing disaccharide units containing *α*, *β*-unsaturated hexuronic acids: ΔDi-0S_HA_, ΔDi-0S_CS_, ΔDi-mono4S, ΔDi-mono6S. Chondroitinase AC II acts on the same bond, but it is not able to cleave glycosidic bond between hexosamine and iduronate (IdoA), so it completely degrades the CS chains and, to some extent, the DS ones. In the present paper, CS is defined as only containing glucuronate, while DS is defined as containing some quantity of iduronate.

Dried GAGs were dissolved in 100 *μ*L of 100 mM ammonium acetate, pH 8.0. Separate digestions with chondroitin ABC and AC II lyases were performed at 37°C for 24 h, using 0.1 U per 100 *μ*g hexuronic acid. The digestion mixture was boiled for 1 min to inactivate the enzyme, centrifuged at 11000 ×g for 5 min, and vacuum-dried. The free reducing groups exposed by enzyme cleavage can be fluorescently tagged with 2-aminoacridone (AMAC) by reductive amination in presence of sodium cyanoborohydride (NaBH_3_CN) [29, 30]. This method allows the labeling of the reducing ends of unsaturated disaccharides obtained after enzymatic degradation of GAG chains, improving dramatically the sensitivity of various analytical techniques used for identification and quantitation of GAGs [30, 31].

Briefly, 40 *μ*L of 12.5 mM AMAC solution in glacial acetic acid/DMSO (3 : 17 v/v) was added to the lyophilized sample aliquots, and samples were incubated for 10–15 minutes at room temperature. Then 40 *μ*L of 1.25 M NaBH_3_CN in ultrapure water was added to each sample, and the mixtures were incubated at 45°C for 4 hours. After derivatization, 20 *μ*L of glycerol (20% final concentration) was added to each sample prior electrophoresis. PAGE was performed according to Karousou et al. [31], in a Mini-Protean II cell vertical slab gel electrophoresis apparatus (Bio-Rad). Electrophoresis was performed in 0.15 M Tris-borate, pH 8.8, at 400 V and 4°C. Gels were scanned in a UV-light box using a CCD camera (Gel Doc XR System) and analyzed with Quantity One 4.6.3 from Bio-Rad Laboratories. For quantitation of Δ-disaccharides, a CS calibration curve was built using commercial chondroitin sulfate A subjected to chondroitin ABC and AC lyase treatment and derivatization procedure.

The ratios of glucuronate to iduronate containing disaccharides and 4-sulfation to 6-sulfation were calculated.

The GAG class levels from each sample as measured by FACE were normalized to the DDT weight to estimate the tissue concentrations.

### 2.5. Isolation and Seeding of Bovine Fibroblasts on TriCol-Treated Pericardium

Bovine fibroblasts were isolated from bovine pericardium by 2 mg/mL collagenase II digestion and cultured in DMEM HEPES (Sigma, St.Louis, USA) modification (10% FBS, 1% glutamine, and 1% penicillin/streptomycin) at 37°C and 5% CO_2_. Cells were left in culture up to the 3rd passage.

Seeding was performed on 1.77 cm^2^ of circle-shaped TriCol-treated pericardium samples in 24-well flat bottom-culture plate (BD, NJ, USA). Suspension of 750,000 cells/cm^2^ in 1 mL DMEM HEPES (Sigma, St.Louis, USA) modification (10% FBS, 1% glutamine, and 1% penicillin/streptomycin) was distributed onto each pericardial sample. After 7 days of culture in incubator, the specimens were collected and processed for histological evaluation.

### 2.6. Histological Staining

To assess the presence, surface spreading and penetration of fibroblasts after seven days in vitro culture on TriCol-treated pericardium, specimens were embedded in Optimal Cutting Temperature Medium (OCT Bioptica, Milano, Italy), sectioned at 8 *μ*m and stained for hematoxylin and eosin (H and E) (Rapid Frozen Section Kit, Bioptica, Milano, Italy).

### 2.7. Mechanical Testing

Stress-strain tests of native and decellularized bovine pericardium were performed in a tensiometer Zwich Z0.5 (Brugger HSG/ETK). For both native and TriCol-treated pericardium, bone-shaped samples were excised from the pericardial area facing the left ventricle anterior wall and with the main axis orthogonal to the interventricular septum. The area of the specimens free from the grips was a rectangle with a length of 34 mm and a width of 6 mm. The thickness of the specimen was measured with a digital thickness gauge and used to calculate the cross-sectional area. The mechanical tests were performed in physiological solution at RT, and for every sample an elongation of 30 mm/m was applied bringing the tissue to rupture. Data of elongation and force applied were collected every second.

### 2.8. Statistical Analysis

Data are reported as mean ± standard deviation or total numbers and relative frequencies. For comparison between two groups, Student's *t*-test was performed. A value of *P* ≤ 0.05 was considered statistically significant. The analyses were performed with Microsoft SPSS 11.0 and SigmaStat 3.11.0 software.

## 3. Results

In the three conduit tissues (leaflet, sinus, and wall) of both NT AR and PR, the differential concentration of total GAGs (normalized to DDT) was in turn deeply different from that in pericardium (Table 1). Particularly, in AR, the GAG content of leaflet and sinus was similar and three times higher than that of arterial wall. However, in PR GAG content of leaflet was twice that found in both sinus and pulmonary arterial wall. Otherwise, by comparing the two valvulated conduits, GAG concentration in AR leaflet resulted twice that in PR leaflet while that in aortic wall was similar to that in pulmonary artery wall. In turn the GAG content of pericardium accounted to about half that in the arterial wall of both valvulated conduits.

TriCol-based decellularization procedure produced a relevant loss of GAGs. Relative to native aortic valve, the total GAG content in TriCol-decellularized specimens was reduced by 72% in leaflet, 69% in sinus, and 52% in arterial wall. For pulmonary valve, total GAG reduction was by 49% in leaflet, 53% in sinus, and 33% in wall. In pericardial samples, decellularization reduced total GAG concentration by 39%.

As already reported in a previous paper [26], GAG distribution was partly different in each different portion (leaflet, sinus, and wall) of NT AR and PR. In both conduits chondroitin sulfate (CS) isomers and hyaluronan were present in the greatest proportion, the hyaluronan exhibiting a decreasing gradient from leaflet to arterial wall. Moreover, in leaflets and sinuses of both valves CS isomers comprised a slow migrating CS, as assessed by acetate cellulose electrophoresis of free intact polysaccharides.

In pericardium, the GAG class present in the greatest proportion was dermatan sulfate (DS), with very low amounts of hyaluronan, as assessed by discontinuous electrophoresis of intact chains (data not shown).

### 3.1. GAG Structural Analysis

**Figure 1:**
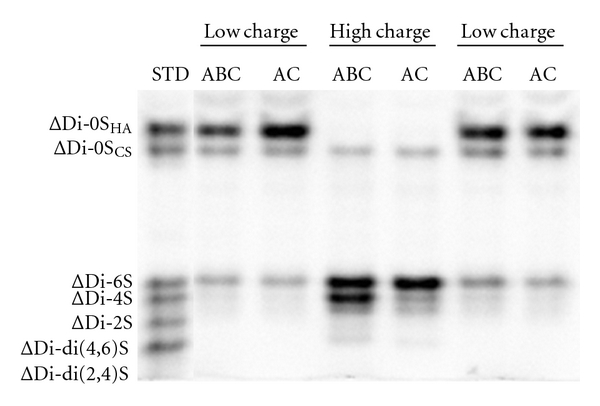
Representative FACE separation of unsaturated AMAC-labeled disaccharides obtained from low- (0.55 M NaCl) and high-charged (1 M NaCl) GAGs after depolymerization with Chase ABC and AC.

**Figure 2:**
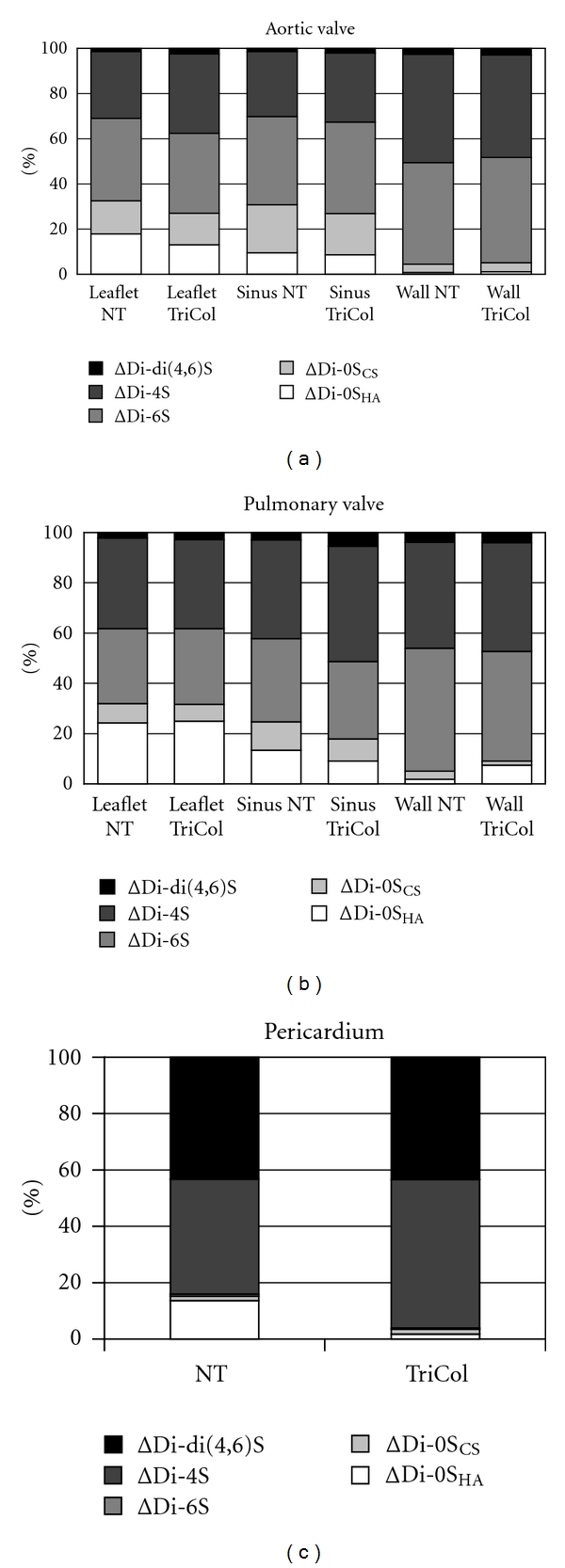
Relative Δ-disaccharides content in fresh (NT) and decellularized (TriCol) porcine heart valves and pericardium, as calculated from FACE data.

Since the relative proportion of HS in sinuses and leaflets of native cardiac valves and in pericardium was very low, we focused on the structural characterization of CS isomers purified from NT and TriCol samples. FACE analysis allows us to detect both mono- and di-sulfated CS-derived disaccharides, also discriminating between the nonsulfated forms released from CS isomers and those from HA (Figure 1). The distributions of Δ-disaccharides in porcine cardiac valves were similar in each selected portion of aortic and pulmonary conduits, while pericardial tissue contained an abundance of ΔDi-mono4S and ΔDi-diS with very low proportions of nonsulfated and 6 sulfated Δ-disaccharides (Figure 2).

There were no significant changes in Δ-disaccharides distributions in each sample following decellularization, even if in aortic leaflet and pericardium the loss seems to affect mainly HA.

**Figure 3:**
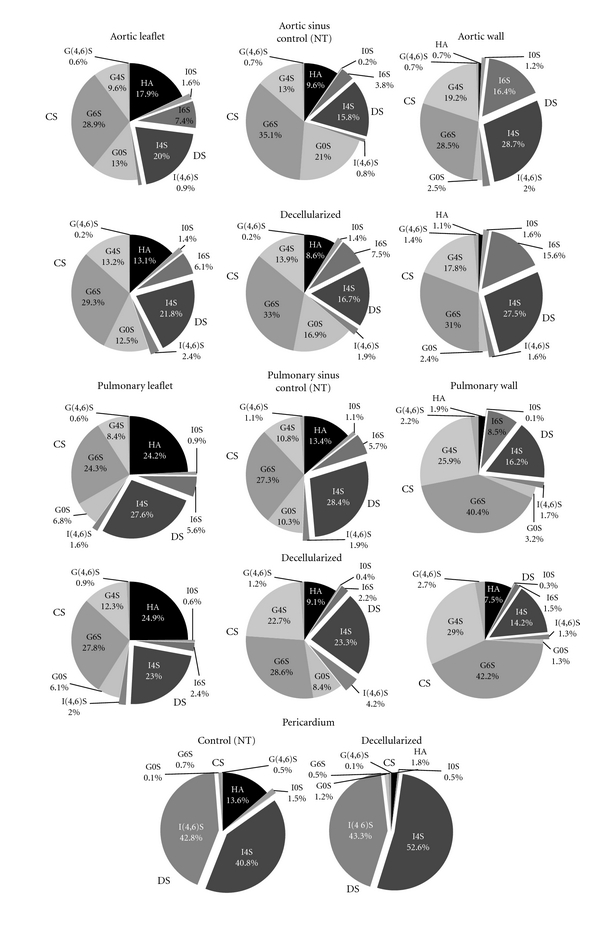
Distribution of glycosaminoglycans (GAGs) in NT and TriCol-treated porcine valves and pericardium, as calculated from FACE data. G0S: nonsulfated GlcA-containing disaccharides, G6S: 6-sulfated GlcA-containing disaccharide, G4S: 4-sulfated GlcA-containing disaccharide, G(4,6)S: disulfated GlcA-containing disaccharide, I0S: nonsulfated IdoA-containing disaccharide, I6S: 6-sulfated IdoA-containing disaccharide, I4S: 4-sulfated IdoA-containing disaccharide, I(4,6)S: disulfated IdoA-containing disaccharide.

The data analyses on ABC/ACII depolymerization revealed that in valve leaflets and sinuses CS was the galactosaminoglycan present in the greatest proportions, while pericardial tissue had almost exclusively DS (Figure 3).

Statistical analysis of the FACE results relative to GalAG structural characterization in native and decellularized valve and pericardium indicated that the depletion in total content does not produce significant changes in the distribution of CS isomers. The percentages of nonsulfated disaccharides from CS isomers are reported in Table 2. Interestingly, significant differences in the under-sulfation degree were detected comparing native aortic and pulmonary valve conduits, referred to leaflet and sinus.

Additional data like epimerization and sulfation patterns of CS isomers were calculated comprehensively on native and treated samples (Table 3). Aortic leaflet and sinus showed a higher proportion of CS respect to the pulmonary ones, a value that was the opposite to that in the artery wall, while in the pericardial tissue, as already stated, the major CS isomer was DS.

### 3.2. Histology

**Figure 4:**
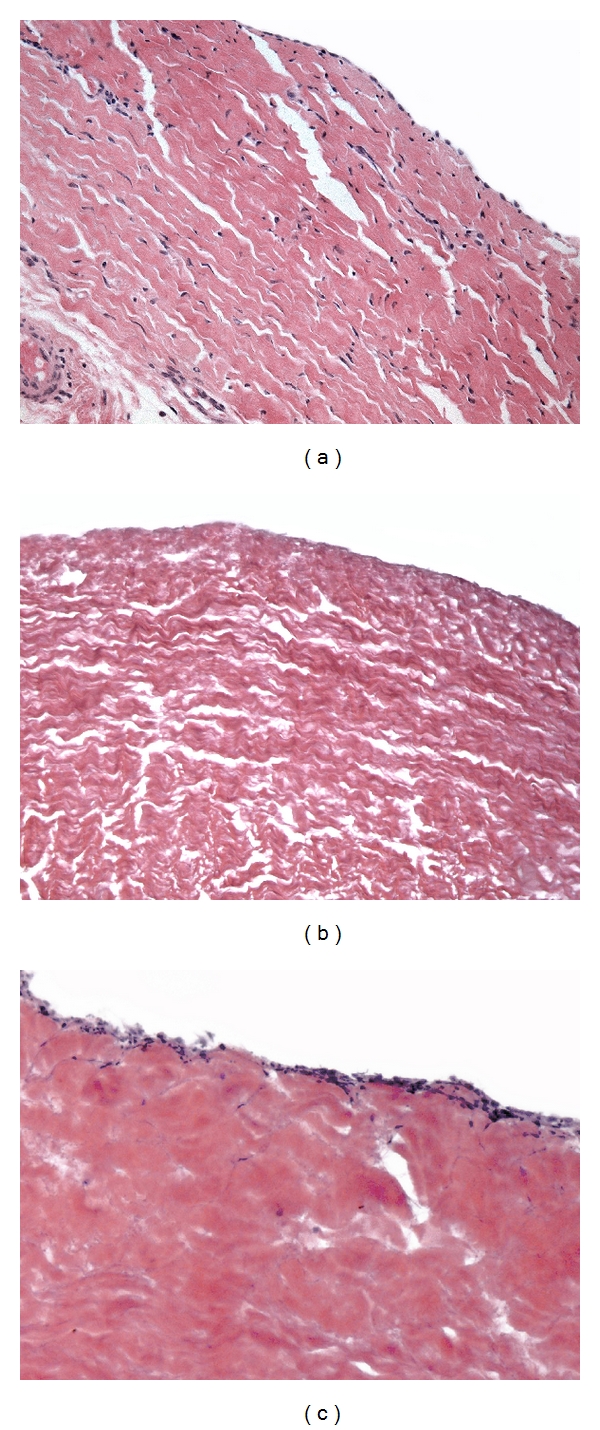
H and E staining of native bovine pericardium (a); TriCol-decellularized bovine pericardium before (b) and after 7 days of bovine fibroblasts seeding (c). Magnification 20x.

H and E staining highlighted the removal of resident cells from bovine pericardium after TriCol treatment (Figure 4(b)) compared to native tissue (Figure 4(a)). The collagen fibers of the resulting matrix exhibited many void areas surrounding the bundles exhibiting a more defined wavy pattern (even if partially shrinked) with respect to the native sample. Seven days after seeding the fibroblasts did adhere to, crowded the pericardial surface and started to colonize the underlying detergent-treated matrix (Figure 4(c)). Moreover, the collagen fiber bundles apparently resumed morphological features approaching those of the native sample.

The propensity of TriCol-treated porcine heart valve leaflet to be repopulated by in vitro seeded cells has been previously reported [27, 32]. 

## 4. Mechanical Testing

**Figure 5:**
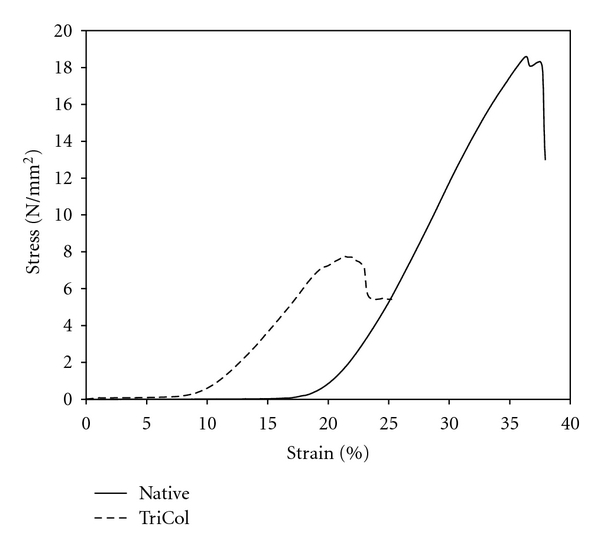
Stress-strain diagram of native and TriCol-decellularized bovine pericardium strips. See Section 2.

A typical stress-strain diagram of native and decellularized pericardial samples is reported in Figure 5. Load-bearing fibrous component of treated pericardium appeared to be tensioned at lower strain with respect to those of native sample as having been partly reoriented in the direction of the stress. Nevertheless in its linear part the slope of the diagram was similar in both preparations even if the load at rupture of TriCol samples accounted to about half that of native ones.

The mechanical properties of TriCol-treated valvular leaflets from pigs have been previously reported [27] to be not significantly different from those of untreated samples although circumferential samples exhibited 20% higher extensibility and trended to lower (about 10%) stiffness.

## 5. Discussion

Recent decellularization studies comprising our combination of ionic and nonionic detergents showed excellent cell removal capacity with preservation of the major structural ECM molecules although ionic detergents like sodium-dodecyl-sulfate (SDS) and sodium deoxycholate (DOC) might somehow modify the resulting scaffold matrix [33, 34]. Particularly, the adopted TriCol procedure is reported to thoroughly remove the *α*-gal antigen responsible for the iperacute rejection of xenogenic grafts and possibly for the chronic inflammation elicited by bioprosthetic devices treated with glutaraldehyde [35].

However, the loss of GAGs following decellularization could have deep effects on the structure of the ECM. In fact, the extraction of the GAGs could affect the mechanical behavior of the valves by introducing flexural rigidity, thickness decrease and favoring an increase in tissue Ca^2+^ content. All of these changes in the ECM composition might lead to problems in valve functionality or directly in valve failure. In this paper, fluorophore-assisted carbohydrate electrophoresis (FACE) was used to provide detailed information regarding GAG distribution as well as quantity and fine structure of various GAGs in porcine vascular tissues (aortic valve, pulmonary valve, and pericardium) used as xenografts for heart valve implants.

This study showed that the content and distribution of GAGs within native aortic valve, pulmonary valve, and pericardium are deeply different. In valve conduits both levels and composition of GAGs were region-specific. The leaflets, which experience compression, contain the highest concentration of GAGs, with an abundance of HA and under-sulfated CS. The quantification and characterization of GAGs in porcine valve conduits by FACE largely agree with our previous results obtained by analyzing intact polysaccharides [26]. The pericardial tissue contains relatively fewer GAGs but higher proportions of dermatan-4-sulfated and dermatan oversulfated. Particularly the level of oversulfation is strikingly higher than that already reported for oversulfated DS from other porcine tissues (e.g., up to 10–20% in skin) which in turn exhibit significant HC-II-mediated inhibition of thrombin activity [36]. Noteworthy, the oversulfation level found here is comparable to that of DS from marine ascidians (50–70%) also studied for its thrombin inhibitory activity [37]. All that could be the rationale behind the still unexplained low thrombogenicity potential of pericardial valve xenografts making the chronic anticoagulation treatment unnecessary in such patients [38]. Moreover, this finding is in good agreement with published data which described the presence of a low-molecular-weight dermatan sulfate proteoglycan in bovine pericardial tissue [39]. The abundance of particular GAGs and PGs can vary according to different biological needs of the tissues. In example, the dermatan sulfate (mostly 4-sulfated) PGs decorin and biglycan regulate the formation and orientation of collagen fibrils and hence tissue tensile strength, whereas the hyaluronan (HA), which is not covalently bound to a core protein, entraps large amounts of water to create a swelling force [40, 41]. It has been speculated that their ability to hydrate the spongiosa layer serves to decrease the shear stresses during valve function, and the presence of negatively charged GAG molecules may reduce calcification by chelating calcium ions, thereby preventing hydroxyapatite nucleation [13, 14, 42]. Moreover, it is thought that decellularization treatment extracts more easily hyaluronan and chondroitin/dermatan-6-sulfate, which exist in tissues as part of the aggregate of hyaluronan and versican PG [43]. These observations suggest that the loss of GAGs may be crucial for the development of new bioprostheses.

Our results showed that there was a relevant extraction of GAGs following TriCol decellularization procedure, but it was not selective, with the only exception of HA from aortic leaflet and pericardium. Distribution of PGs and GAGs in vascular tissue has been reported to be complex, district- and layer-specific, associated with different mechanical environments [11], and could have important implications for heart valve tissue engineering and bioprosthesis development. Although some of these compositional differences may appear quite subtle, such as the degree and position of sulfate and the degree and position of 5′epimerization on sulfated GAG chains, these fine structural distinctions may have important biological roles such as binding sites for other matrix components or for signaling cell differentiation in addition to their possible influence on the scaffold thrombogenicity. The possibility to assess carefully the ΔDi-mono4S and ΔDi-mono6S content in tissues is important considering that modifications of the ΔDi-mono4S/Δdi-mono6S ratio and the ratio of nonsulfated to sulfated GAGs have been described in aging and disease. The C4S/C6S ratio has important biological implication; in humans, it decreases progressively in pulmonary artery, iliac artery, and aorta [44]. Moreover, the relative content of C4S decreases about fivefold in human aorta [45] and cerebral arteries [46] with aging and C4S/C6S ratio decreases particularly with atherosclerosis development [47].

Furthermore, this ratio might gather more information about the mechanical behavior and hence, be used to hypothesize the type of PGs found in the valves associated to GAG class concentrations and fine structure characteristics [10, 11].

H and E staining showed that TriCol procedure did in fact remove the cells from the native pericardium while the resulting matrix revealed to be suitable for cell repopulation. The fibroblasts adhered to the pericardial surface and started, after 7-day culture, to spread into the underlying extracellular matrix. Removal of cells did modify partly the mechanics of the native pericardium while being otherwise compatible with the features expected for the making of valve substitutes although the ultimate mechanical behaviour of repopulated scaffolds has still to be explored. As a whole both TriCol-decellularized porcine heart valves and bovine pericardium appeared a promising material bearing the potential for future development of tissue-engineered heart valve scaffolds able to be recellularized by the patient own cells.

Future studies are expected to examine the inherent complexity within valve tissues, due to the histological layers, mechanical forces, matrix composition and effects of aging, and also the functional characteristic of different GAGs that could have impact on the function of normal valves and on the choice of the candidate cardiovascular tissue to produce the best scaffold for the development of a tissue-engineered heart valve.

## Figures and Tables

**Table 1:** Total GAG content (*μ*g hexuronate/mg DDT) in the selected areas of cardiac valves and in pericardial tissue (before and after TriCol treatment).

	Aortic valve			Pulmonary valve			Pericardium
	Leaflet	Sinus	Wall	Leaflet	Sinus	Wall	
NT	17.50 ± 5.55	18.21 ± 11.57	5.74 ± 3.72	9.11 ± 5.02	5.66 ± 0.94	4.18 ± 1.09	2.34*
TriCol	4.88 ± 3.43	5.61 ± 2.16	2.74 ± 0.60	4.61 ± 1.23	2.62 ± 0.95	2.82 ± 0.87	1.44*

*pooled samples.

**Table 2:** Percentages of nonsulfated disaccharides from CS isomers in the examined tissues (means ± SD). Significant differences (*P* < 0.05) are reported in bold.

ΔDi-nonsulfated/total Δ-disaccharides			
	NT	TriCol	

Pericardium	0.019	0.017	

Aortic valve			
	NT	TriCol	*P*

Leaflet	0.177 ± 0.031	0.150 ± 0.024	0.228
Sinus	0.237 ± 0.031	0.158 ± 0.056	0.061
Artery wall	0.028 ± 0.016	0.048 ± 0.040	0.404

Pulmonary valve			
	NT	TriCol	*P*

Leaflet	0.100 ± 0.017	0.090 ± 0.020	0.463
Sinus	0.129 ± 0.021	0.086 ± 0.029	0.070
Artery wall	0.029 ± 0.019	0.018 ± 0.004	0.380

	Aortic versus pulmonary		
	NT	TriCol	

Leaflet	**0.005**	**0.008**	
Sinus	**0.001**	0.117	
Artery wall	0.938	0.274	

**Table 3:** Levels of epimerization and sulfation of CS isomers (means ± SD).

	CS/DS	CS	DS	Total GalAGs (CS + DS)
Aortic valve	GlcA/IdoA	4S/6S	4S/6S	4S/6S
Leaflet	1.666 ± 0.645	0.375 ± 0.072	2.340 ± 0.699	0.823 ± 0.121
Sinus	2.611 ± 0.908	0.397 ± 0.072	2.359 ± 1.175	0.724 ± 0.077
Artery wall	1.446 ± 0.843	0.644 ± 0.111	2.221 ± 0.910	0.971 ± 0.074

Pulmonary valve				

Leaflet	1.180 ± 0.404	0.412 ± 0.087	2.605 ± 0.759	1.304 ± 0.525
Sinus	1.476 ± 0.673	0.589 ± 0.429	3.995 ± 0.665	1.273 ± 0.190
Artery wall	3.133 ± 0.777	0.681 ± 0.103	1.982 ± 0.760	0.905 ± 0.074

Pericardium*	0.017	*	*	80.289

*Single data regarding C4S/C6S and D4S/D6S for pericardium were very high, as 6-sulfation was near to 0.

## References

[1] Human P, Zilla P (2001). Inflammatory and immune processes: the neglected villain of bioprosthetic degeneration?. *Journal of Long-Term Effects of Medical Implants*.

[2] Briand M, Pibarot P, Després JP (2006). Metabolic syndrome is associated with faster degeneration of bioprosthetic valves. *Circulation*.

[3] Flanagan TC, Pandit A (2003). Living artificial heart valve alternatives: a review. *European Cells and Materials*.

[4] Goldbarg SH, Elmariah S, Miller MA, Fuster V (2007). Insights into degenerative aortic valve disease. *Journal of the American College of Cardiology*.

[5] Vesely I (2005). Heart valve tissue engineering. *Circulation Research*.

[6] Sacks MS, Schoen FJ, Mayer JE (2009). Bioengineering challenges for heart valve tissue engineering. *Annual Review of Biomedical Engineering*.

[7] Rothenburger M, Volker W, Vischer P, Glasmacher B, Scheld HH, Deiwick M (2002). Ultrastructure of proteoglycans in tissue-engineered cardiovascular structures. *Tissue Engineering*.

[8] Grande-Allen KJ, Clabro A, Gupta V, Wight TN, Hascall VC, Vesely I (2004). Glycosaminoglycans and proteoglycans in normal mitral valve leaflets and chordae: association with regions of tensile and compressive loading. *Glycobiology*.

[9] Stephens EH, Chu CK, Grande-Allen KJ (2008). Valve proteoglycan content and glycosaminoglycan fine structure are unique to microstructure, mechanical load and age: relevance to an age-specific tissue-engineered heart valve. *Acta Biomaterialia*.

[10] Kinsella MG, Bressler SL, Wight TN (2004). The regulated synthesis of versican, decorin, and biglycan: extracellular matrix proteoglycans that influence cellular phenotype. *Critical Reviews in Eukaryotic Gene Expression*.

[11] Taylor KR, Gallo RL (2006). Glycosaminoglycans and their proteoglycans: host-associated molecular patterns for initiation and modulation of inflammation. *FASEB Journal*.

[12] Schoen FJ, Levy RJ (1999). Tissue heart valves: current challenges and future research perspectives. *Journal of Biomedical Materials Research*.

[13] Vyavahare NR, Ogle M, Schoen FJ (1999). Mechanisms of bioprosthetic heart valve failure: fatigue causes collagen denaturation and glycosaminoglycan loss. *Journal of Biomedical Materials Research*.

[14] Lovekamp JJ, Simionescu DT, Mercuri JJ, Zubiateb B, Sacksb MS, Vyavahare NR (2006). Stability and function of glycosaminoglycans in porcine bioprosthetic heart valves. *Biomaterials*.

[15] Simionescu DT, Lovekamp JJ, Vyavahare NR (2003). Glycosaminoglycan-degrading enzymes in porcine aortic heart valves: implications for bioprosthetic heart valve degeneration. *Journal of Heart Valve Disease*.

[16] Simionescu DT, Lovekamp JJ, Vyavahare NR (2003). Degeneration of bioprosthetic heart valve cusp and wall tissues is initiated during tissue preparation: an ultrastructural study. *Journal of Heart Valve Disease*.

[17] Grande-Allen KJ, Mako WJ, Calabro A, Shi Y, Ratliff NB, Vesely I (2003). Loss of chondroitin 6-sulfate and hyaluronan from failed porcine bioprosthetic valves. *Journal of Biomedical Materials Research A*.

[18] Grande-Allen KJ, Osman N, Ballinger ML, Dadlani H, Marasco S, Little PJ (2007). Glycosaminoglycan synthesis and structure as targets for the prevention of calcific aortic valve disease. *Cardiovascular Research*.

[19] Dainese L, Barili F, Topkara VK (2006). Effect of cryopreservation techniques on aortic valve glycosaminoglycans. *Artificial Organs*.

[20] Torii S, Banshey RI, Nakao K (1965). Acid Mucopolysaccharide composition of human heart valve. *Biochim Biophys Acta*.

[21] Moretti A, Whitehouse MW (1963). Changes in the mucopolysaccharide composition of bovine heart valves with age. *Biochemical Journal*.

[22] Sell S, Scully RE (1965). Aging changes in the aortic and mitral valves: histologic and histochemical studies with observations on calcific aortic stenosis and calcification of the mitral annulus. *The American Journal of Pathology*.

[23] Kuschen GS, Coulin F, Power CA (1999). Glycosaminoglycans interact selectively with chemokines and modulate receptor binding and cellular responses. *Biochemistry*.

[24] Linhardt RJ, Toida T (2004). Role of glycosaminoglycans in cellular communication. *Accounts of Chemical Research*.

[25] Wille I, Rek A, Krenn E, Kungl A (2007). Biophysical investigation of human heparin sulfate D-glucosaminyl 3-O-sulfotransferase-3A: a mutual effect of enzyme oligomerisation and glycosaminoglycan ligand binding. *Biochim Biophys Acta*.

[26] Naso F, Gandaglia A, Formato M (2010). Differential distribution of structural components and hydration in aortic and pulmonary heart valve conduits: impact of detergent-based cell removal. *Acta Biomaterialia*.

[27] Spina M, Ortolani F, Messlemani AE (2003). Isolation of intact aortic valve scaffolds for heart-valve bioprostheses: extracellular matrix structure, prevention from calcification, and cell repopulation features. *Journal of Biomedical Materials Research A*.

[28] Bitter T, Muir HM (1962). A modified uronic acid carbazole reaction. *Analytical Biochemistry*.

[29] Calabro A, Benavides M, Tammi M, Hascall VC, Midura RJ (2000). Microanalysis of enzyme digests of hyaluronan and chondroitin/dermatan sulfate by fluorophore-assisted carbohydrate electrophoresis (FACE). *Glycobiology*.

[30] Zinellu A, Pisanu S, Zinellu E (2007). A novel LIF-CE method for the separation of hyalurnan- and chondroitin sulfate-derived disaccharides: application to structural and quantitative analyses of human plasma low- and high-charged chondroitin sulfate isomers. *Electrophoresis*.

[31] Karousou EG, Militsopoulou M, Porta G, De Luca G, Hascall VC, Passi A (2004). Polyacrylamide gel electrophoresis of fluorophore-labeled hyaluronan and chondroitin sulfate disaccharides: application to the analysis in cells and tissues. *Electrophoresis*.

[32] Iop L, Renier V, Naso F (2009). The influence of heart valve leaflet matrix characteristics on the interaction between human mesenchymal stem cells and decellularized scaffolds. *Biomaterials*.

[33] Korossis SA, Booth C, Wilcox HE (2002). Tissue engineering of cardiac valve prostheses II: biomechanical characterization of decellularized porcine aortic heart valves. *Journal of Heart Valve Disease*.

[34] Crapo PM, Gilbert TW, Badylak SF (2011). An overview of tissue and whole organ decellularization processes. *Biomaterials*.

[35] Naso F, Gandaglia A, Iop L, Spina M, Gerosa G (2011). First quantitative assay of alpha-Gal in soft tissues: presence and distribution of the epitope before and after cell removal from xenogeneic heart valves. *Acta Biomaterialia*.

[36] Bartolucci C, Cellai L, Iannelli MA (1995). Inhibition of human leukocyte elastase by chemically and naturally oversulfated galactosaminoglycans. *Carbohydrate Research*.

[37] Kozlowski EO, Lima PC, Vicente CP (2011). Dermatan sulfate in tunicate phylogeny: order-specific sulfation pattern and the effect of [→4IdoA(2-Sulfate)*β* − 1→3GalNAc(4-Sulfate)*β* − 1→] motifs in dermatan sulfate on heparin cofactor II activity. *BMC Biochemistry*.

[38] Garcia-Bengoecbea JB, Gonzilez-Juanatey JR, Rubio J, DurHn D, Sierra J (1991). Thromboembolism in patients with pericardial valves in the absence of chronic anticoagulation: 12 years’ experience. *European Journal of Cardio-Thoracic Surgery*.

[39] Simionescu D, Iozzo RV, Kefalides NA (1989). Bovine pericardial proteoglycan: biochemical, immunochemical and ultrastructural studies. *Matrix*.

[40] Liao J, Joyce EM, Sacks MS (2008). Effects of decellularization on the mechanical and structural properties of the porcine aortic valve leaflet. *Biomaterials*.

[41] Gupta V, Werdenberg JA, Lawrence BD, Mendez JS, Stephens EH, Grande-Allen KJ (2008). Reversible secretion of glycosaminoglycans and proteoglycans by cyclically stretched valvular cells in 3D culture. *Annals of Biomedical Engineering*.

[42] Shah SR, Vyavahare NR (2008). The effect of glycosaminoglycan stabilization on tissue buckling in bioprosthetic heart valves. *Biomaterials*.

[43] Gupta V, Barzilla JE, Mendez JS (2009). Abundance and location of proteoglycans and hyaluronan within normal and myxomatous mitral valves. *Cardiovascular Pathology*.

[44] Cardoso LEM, Mourao PAS (1994). Glycosaminoglycan fractions from human arteries presenting diverse susceptibilities to atherosclerosis have different binding affinities to plasma LDL. *Arteriosclerosis and Thrombosis*.

[45] Toledo OMS, Mourao PAS (1979). Sulfated glycosaminoglycans of human aorta: chondroitin 6-sulfate increase with age. *Biochemical and Biophysical Research Communications*.

[46] Murata K, Yokoyama Y (1989). Acidic glycosaminoglycans in human atherosclerotic cerebral arterial tissues. *Atherosclerosis*.

[47] Theocharis AD, Theocharis DA, De Luca G, Hjerpe A, Karamanos NK (2002). Compositional and structural alterations of chondroitin and dermatan sulfates during the progression of atherosclerosis and aneurysmal dilatation of the human abdominal aorta. *Biochimie*.

